# Global Expression of Cell Surface Proteins in Embryonic Stem Cells

**DOI:** 10.1371/journal.pone.0015795

**Published:** 2010-12-29

**Authors:** Bin Gu, Jiarong Zhang, Wei Wang, Lijuan Mo, Yang Zhou, Liangbiao Chen, Yusen Liu, Ming Zhang

**Affiliations:** 1 The Institute of Cell Biology and Genetics, College of Life Sciences, Zhejiang University, Hangzhou, China; 2 The Institute of Genetics and Developmental Biology, Chinese Academic of Sciences, Beijing, China; 3 Center for Perinatal Research, The Research Institute at Nationwide Children's Hospital, Department of Pediatrics, The Ohio State University College of Medicine, Columbus, Ohio, United States of America; Institute of Molecular Genetics, CNRS, France

## Abstract

**Background:**

Recent studies have shown that embryonic stem (ES) cells globally express most genes in the genome at the mRNA level; however, it is unclear whether this global expression is propagated to the protein level. Cell surface proteins could perform critical functions in ES cells, so determining whether ES cells globally express cell surface proteins would have significant implications for ES cell biology.

**Methods and Principal Findings:**

The surface proteins of mouse ES cells were purified by biotin labeling and subjected to proteomics analysis. About 1000 transmembrane or secreted cell surface proteins were identified. These proteins covered a large variety if functional categories including signal transduction, adhesion and transporting. More over, mES cells promiscuously expressed a wide variety of tissue specific surface proteins. And many surface proteins were expressed heterogeneously on mES cells. We also find that human ES cells express a wide variety of tissue specific surface proteins.

**Conclusions/Significance:**

Our results indicate that global gene expression is not simply a result of leaky gene expression, which could be attributed to the loose chromatin structure of ES cells; it is also propagated to the functional level. ES cells may use diverse surface proteins to receive signals from the diverse extracellular stimuli that initiate differentiation. Moreover, the promiscuous expression of tissue specific surface proteins illuminate new insights into the strategies of cell surface marker screening.

## Introduction

Embryonic stem (ES) cells are pluripotent stem cells from early embryos [1], [2]. It has been proposed that the maintenance of their self- renewal capacity depends on the sustained expression of ES-specific genes like Oct4 and Nanog and the suppressed expression of differentiation-associated genes [3], [4], [5]. However, recent studies have shown that ES cells possess a loose chromatin structure [6], [7], [8], and most genes in the genome of ES cells are associated with activating epigenetic modifications and are expressed at low levels as transcripts [9], [10]. Moreover, Nishikawa et al. and our group have shown that the core regulator Aire, which promotes the promiscuous expression of tissue-specific genes in the thymus, is expressed in ES cells and induced pluripotent stem cell(iPS) cells [11], [12]. With these findings, the phenomenon that ES cells globally express genes on the mRNA level seems to be well established. However, whether this global expression is just leaky transcription (as a consequence of loose chromatin), or has an actual functional significance, is an issue of debate. Proteins are the functional entities of genes, so determining whether ES cells globally express genes at the protein level would help to resolve the debate and elucidate the biological significance of global gene expression.

Embryonic stem cells depend on specific extracellular signals, like LIF signaling, and metabolites, like threonine, to maintain their self-renewal capacity [13], [14]. ES cells also depend on extracellular signals to initiate their differentiation [15]. Cell surface proteins mediate the interaction of ES cells with extracellular factors, making them an important functional group in ES cells. Moreover, cell surface proteins are candidates for use as specific markers in screening [16]. Therefore, exploring the pattern of cell surface protein expression on ES cells is important for understanding the mechanisms of ES cell self-renewal and differentiation and can help to establish strategies for surface marker discovery.

Proteomics technologies allow for the large-scale scanning of proteins. However, because a significant fraction of cell surface proteins are transmembrane and have a relatively low abundance and solubility [17], differential extraction is required to reduce the abundance range and the complexity of the samples to acquire good quality results. Cell surface labeling and affinity purification is a standard method to selectively extract cell surface proteins [18].

In this study, we labeled the surface proteins of mouse ES (mES) cells with membrane-impermeable biotins and then purified the proteins by streptavidin affinity purification. The purified proteins were analyzed by LC-MS/MS, and 991 cell surface proteins were identified. Bioinformatics studies showed that mES cells expressed a large variety of cell surface proteins with a broad range of functions and tissue distributions. The results were further confirmed by several biochemical methods. Moreover, we showed that hES cells also expressed a variety of tissue-specific surface proteins. Our results demonstrate that the global gene expression in ES cells is propagated to the protein level, which may have a functional significance. Moreover, we propose that new strategies should be implemented to screen for specific surface markers of ES cells.

## Results

### Proteomics analysis of cell surface proteins on mES cells

**Figure 1:**
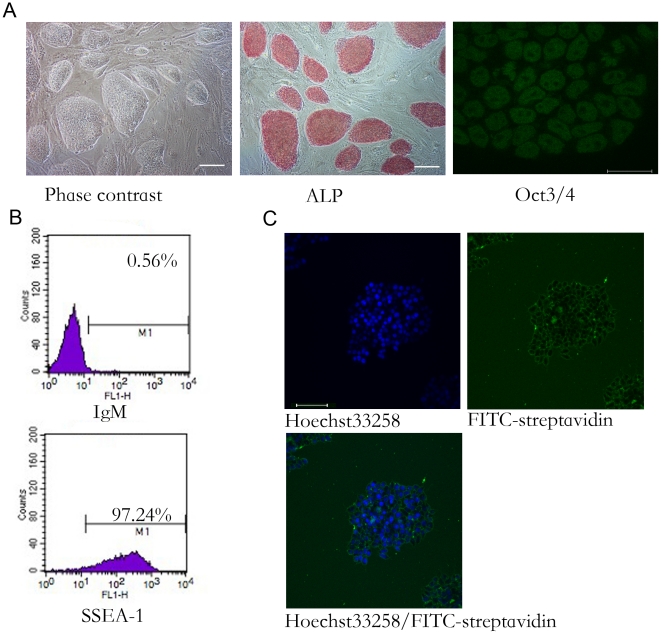
Labeling of mES cell surface proteins. A. Undifferentiated state of mES cells used to purify cell surface proteins. Left panel. Morphology of mES cells used in this study. The undifferentiated mES cells grew as compact colonies. Middle panel. mES cells expressed Alkaline phospatase (ALP). Right panel, Immunocytochemistry staining showed that mES cells expressed Oct4. The bars in left and middle panel represented 100 um while the bar in the right panel represented 20 um. B. Flow cytometry showed that most mES cells used in this study expressed the ES specific surface marker SSEA-1. FITC-streptavidin staining showed that most biotins were labeled on the cell surface.

To explore the expression pattern of ES cell surface proteins, we extracted mES cell surface proteins by biotin labeling and performed protein identification by LC-MS/MS. Before labeling, the quality of the mES cells was evaluated. As shown in Figure 1A, the mES cells used in this study grew with typical colony morphology and homogeneously expressed alkaline phosphatase (ALP) and Oct4. Quantitative analysis by flow cytometry showed that more than 97% of the cells were positive for SSEA-1. These data demonstrate that most mES cells used in this study were undifferentiated. The surface proteins of the mES cells were then labeled with membrane-impermeable biotin reagents and the labeling efficiency was monitored by streptavidin-FITC staining. As shown in Figure 1C, most of the cells were labeled with biotin on the cell surface, although some intracellular labeling could be observed, which could be explained by the staining of apoptotic cells that are common in mES populations.

**Figure 2:**
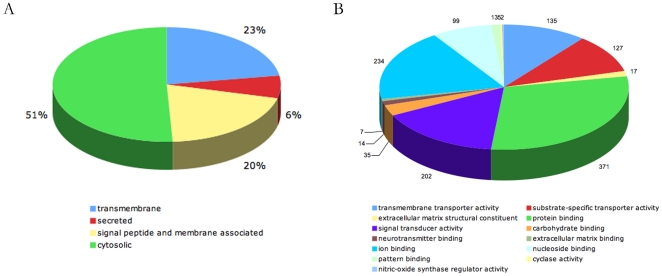
Proteomics analysis of mES cell surface proteins. A. Subcellular distribution of proteins identified in this study. B. Molecular Function categorization of mES cell surface proteins identified in this study.

The biotin-labeled proteins were resolved by SDS-PAGE and analyzed by LC-MS/MS. A total of 3468 proteins were identified. The transmembrane structure and signal peptides were predicted using SOSUI software[19]. Proteins annotated as ‘membrane’ in gene ontology or those predicted to contain transmembrane domains or signal peptides were annotated as general membrane proteins. Of the identified proteins, 1699 were annotated as general membrane proteins, of which 778 were integral membrane proteins with a transmembrane domain or a lipid anchorage, 213 were secreted proteins and 698 were membrane-associated proteins (Figure 2A). Therefore, about half of the identified proteins were general membrane proteins, which is consistent with other reports that used the same methods. We selected the integral membrane proteins and secreted proteins that adhere to the cell surface as cell surface proteins and performed further analysis (Table S1). We first evaluated the expression of 350 randomly selected surface proteins by RT-PCR, and 274 of them were confirmed to be expressed on mES cells. Therefore, our results should be at least 75% accurate. We performed a gene ontology analysis according to the Molecular Function annotations. As shown in Figure 2B, the cell surface proteins of mES cells perform a wide variety of molecular functions. Moreover, each functional category included many functional surface proteins. For example, many different signaling receptors from different pathways were identified (discussed below). Diverse adhesion molecules, including types of cadherins/protocadherins, cell adhesion molecules, and integrins were identified in this study. Various transporting proteins were also identified, including 50 types of channel proteins and 66 types of transporter proteins. Among these were 12 types of ABC-type ATPases from five different families. Diverse extracellular matrix proteins were also identified. Moreover, 180 uncharacterized cell surface proteins were identified, which could serve as candidates for surface marker screening.

### mES Cells express diverse signaling molecules

As signal transduction processes critically regulate the self-renewal and differentiation initiation of ES cells, we analyzed the signaling receptors and ligands that we identified on ES cells. Signaling molecules from 50 distinct signaling pathways were presented on the cell surface of mES cells, among which were receptors and ligands. Some of the signaling pathways like the LIF pathway, BMP pathway, and Wnt pathway have been extensively characterized as critical for the self-renewal of ES cells [13], [20], [21]. Other pathways, like the GABA, acetylcholine, Toll-like receptor and PTP pathways have been proposed to be functional in the self-renewal and proliferation of ES cells according to some reports [22], [23], [24], [25]. However, most of the signaling receptors and ligands identified in this study, like the Eph pathways, semaphorin pathways, olfactory receptor pathways and vomeronasal receptor pathways, have never been functionally characterized in ES cells. These data indicate that mES cells possess a much more versatile signal processing ability than previously thought.

**Figure 3:**
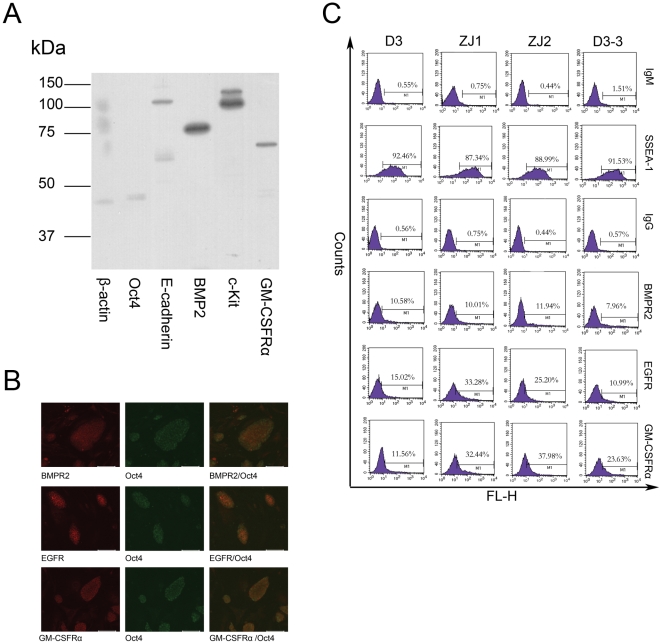
Cell surface signal molecules expressed on mES cells. A. Western blotting showed that mES cells expressed BMP2, c-Kit and GM-CSFRa along with mES specific marker Oct4 and mES surface protein E-cadherin. B. Immunocytochemistry staining showed that mES cells expressed BMPR2, EGFR and GM-CSFRα. Left panel, ICC staining of the cell surface signal molecules on mES cells. Middle panel, ICC staining of Oct4 on mES cells. Right panel, co-staining of the cell surface signal molecules and Oct4 on mES cells. The bar represented 100 um. C. Flow cytometry analysis showed that mES cells heterogeneously expressed BMPR2, EGFR and GM-CSFRa. IgM, IgM control (For SSEA-1 staining). IgG, IgG control(For cell surface signal molecules staining). D3, mES D3 cell line. ZJ1, mES ZJ1 cell line. ZJ2, mES ZJ2 cell line. D3-2, a single cell derived cell line derived from D3.

Besides the large-scale proteomics study, we also studied the expression of cell surface signaling molecules on mES cells using antibodies. As shown in Figure 3A, the expression of BMP2, c-Kit, and GM-CSFRα could be detected in mES cells by western blotting. The detection of Oct4 indicated that the mES used in this experiment is undifferentiated and the detection of E-cadherin indicated that membrane proteins were successfully extracted in this experiment.

To detect the cell surface signaling molecules in situ, we performed immunocytochemistry analysis. To rule out the possibility that some signaling molecules were expressed on spontaneously differentiated mES cells, we co-stained with Oct4 and the signaling molecules. As shown in Figure 3B, mES cells expressed EGFR, BMPR2 and GM-CSFRα on the cell surface. The Oct4 staining demonstrated that most cells expressing the signaling molecules were undifferentiated mES cells. Another interesting phenomenon is that the staining of signaling molecules on mES cells was heterogeneous, the fluorescence strength of varied between Oct4 positive cells. To further confirm the heterogeneous expression of cell surface signaling molecules in mES populations, we performed a flow cytometry analysis. As shown in Figure 3C, mES cells showed a heterogeneous expression for BMPR2, GM-CSFRα and EGFR. Although the whole fluorescent peak moved to the right, indicating positive staining. Only a fraction of mES D3 cells strongly expressed BMPR2, GM-CSFRα and EGFR (approximately 10% for BMPR2, 11% for GM-CSFRα and 15% for EGFR). About 90% of the cells stained positive for SSEA-1, indicating undifferentiated state. However, even for SSEA-1, the fluorescent level varied widely. Therefore, mES cells not only expressed signal molecules but also surface markers heterogeneously. To rule out the possibility that the heterogeneous expression was due to the in vitro culture features of this specific cell line, we performed the same analysis on two other ES cell lines established in our Lab, ZjuJ1 and ZjuJ2. As shown in Figure 3C, both lines expressed BMPR2, GM-CSFRa and EGFR, although the percentage of cells that strongly expressed them was different from the D3 cell line. Previous reports have shown that the heterogeneous expressions of some genes like Nanog, Rex1 and Stella in ES populations are subject to epigenetic regulation and have equilibrium properties [26], [27], [28]. To determine whether this also held true for cell surface signaling molecules, we isolated single cell clonal cell lines from mES D3. We analyzed four single cell clonal cell lines and got similar results, therefore only one is shown as a representative. As shown in Figure 3C, the cell lines established from single mES cells also heterogeneously expressed BMPR2, GM-CSFRa and EGFR, while the percentage of cells strongly expressing them was different from the parental D3 cell line. These results indicated that mES cells heterogeneously expressed cell surface signaling molecules and argued for a stochastic mechanism for the regulation of their expressions.

### mES Cells globally express tissue-specific surface proteins

**Figure 4:**
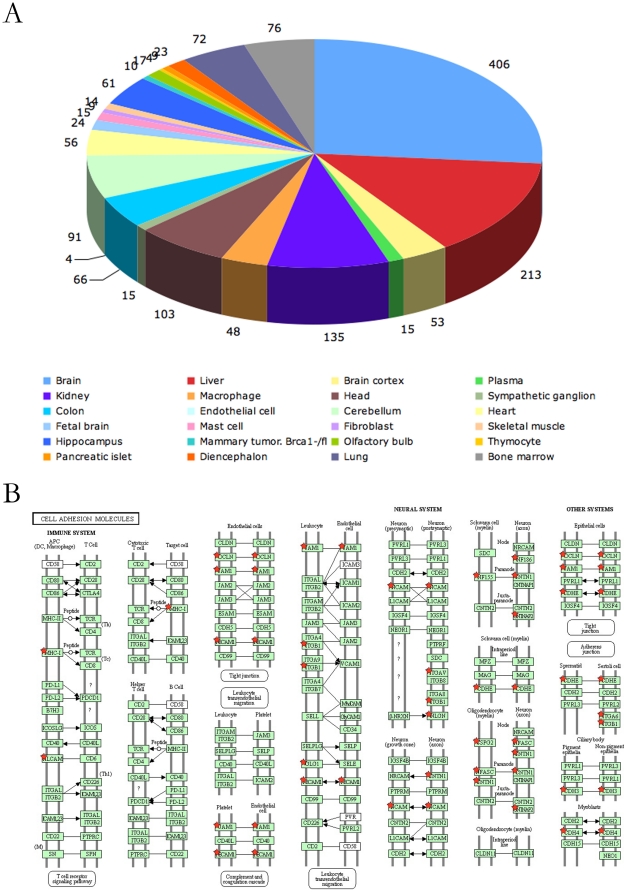
Analysis of tissue specific surface proteins on mES cells. A. mES cells expressed tissue specific surface proteins of a variety of tissue types. B. mES cells expressed a variety of tissue specific cell adhesion molecules. Stars indicated proteins identified in this study.

It has been proposed that many tissue-specific genes are set in a transcriptionally poised state and are expressed at low levels in ES cells [9], [29]. Moreover, others and we have shown that mES cells express the Aire gene, the major regulator of the promiscuous expression of tissue-specific antigens in medullary thymic epithelial cells (mTECs) [11], [12]. Therefore, we set out to determine whether mES cells globally expressed tissue-specific cell surface proteins. We analyzed the surface proteins of mES cells according to the Uniprot tissue specificity annotations using the DAVID software [30], [31]. Of the 991 surface proteins, 904 were annotated as tissue-specific. As shown in Figure 4A, tissue-specific surface proteins from a broad variety of tissue types originated from all three germ layers were expressed in the mES cells. Clonal growth is a basic property of pluripotent cells including mES cells. Cell adhesion molecules plays critical roles in the formation of colonies[32]. As many cell adhesion molecules are tissue specific, we analyzed the cell adhesion molecule pathways using David software. As shown in Figure 4B, mES cells expressed adhesion molecules that function in different cell types including neural cells, epithelia cells, immune cells and germ cells. Considering our data could not cover all the cell surface proteins in mES cells, many other tissue specific cell adhesion molecules should be expressed in mES cells.

**Figure 5:**
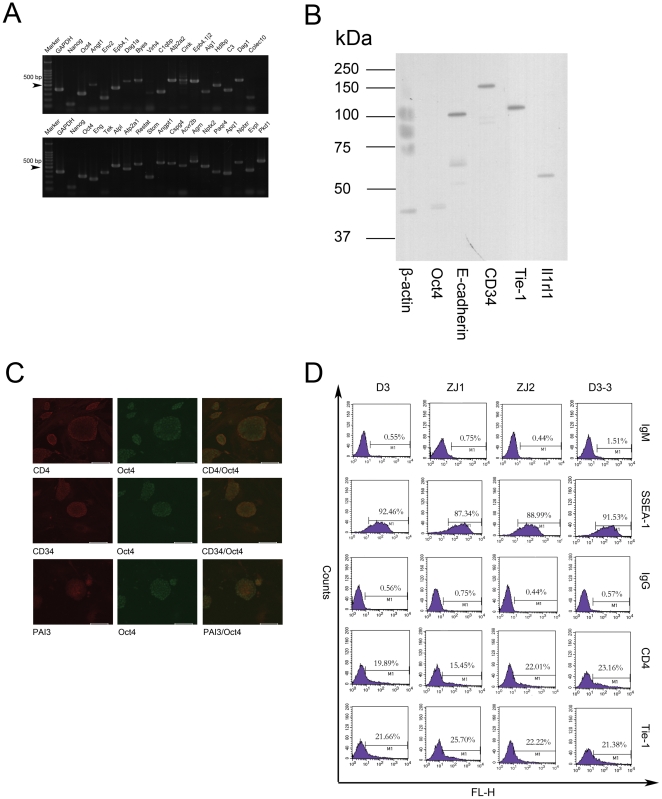
Tissue specific surface proteins expressed on mES cells. A. RT-PCR analysis demonstrated that mES cells expressed tissue specific cell surface proteins. B. Western blotting showed that mES cells expressed CD34, Tie-1 and Il1rl1 along with mES specific marker Oct4 and mES surface protein E-cadherin. C. Immunocytochemistry staining showed that mES cells expressed CD4, CD34 and PAI3. Left panel, ICC staining of the tissue specific cell surface proteins on mES cells. Middle panel, ICC staining of Oct4 on mES cells. Right panel, co-staining of the tissue specific cell surface proteins and Oct4 on mES cells. The bar represented 100 um. D. Flow cytometry analysis showed that mES cells heterogeneously expressed CD4 and Tie-1. IgM, IgM control (For SSEA-1 staining). IgG, IgG control(For tissue specific cell surface proteins staining). D3, mES D3 cell line. ZJ1, mES ZJ1 cell line. ZJ2, mES ZJ2 cell line. D3-2, a single cell derived cell line derived from D3. (Same controls and SSEA-1 staining as in Figure 3 were used since the datas shown here were generated in the same experiment).

We confirmed the expression of some tissue-specific surface proteins by RT-PCR, ICC and western blotting. As shown in Figure 5A, 23 types of tissue-specific surface proteins were confirmed to be expressed in mES cells at the mRNA level. Western blotting further confirmed that hematopoietic stem cell specific-protein CD34, T cell-specific protein CD4, endothelium-specific protein Tie-1 and leukocyte specific protein Il1rl1 were expressed in mES cells (Figure 5B). Moreover, ICC staining showed that mES cells expressed the T-cell-specific CD4 protein, the hematopoietic stem cell-specific CD34 protein and the liver-specific PAI3 protein (Figure 5C). Co-staining with Oct4 demonstrated that the tissue-specific surface proteins were expressed on undifferentiated mES cells. Heterogeneous expression could also be seen from ICC staining. To test whether mES cells heterogeneously express tissue-specific surface proteins like signaling molecules, we performed flow cytometry analysis. As shown in Figure 5D, mES D3 cells expressed CD4 and Tie-1 heterogeneously, only a fraction of the cells strongly expressed the two proteins. Similar to signaling molecules, the heterogeneous expression of tissue-specific surface proteins was consistent between the different mES cells lines and the single cell clonal mES cell lines, while the exact percentage of cells strongly expressing each protein was different. These results further suggest that stochastic mechanisms regulate the tissue-specific surface proteins expression in mES cells.

### hES Cells express tissue-specific surface proteins

**Figure 6:**
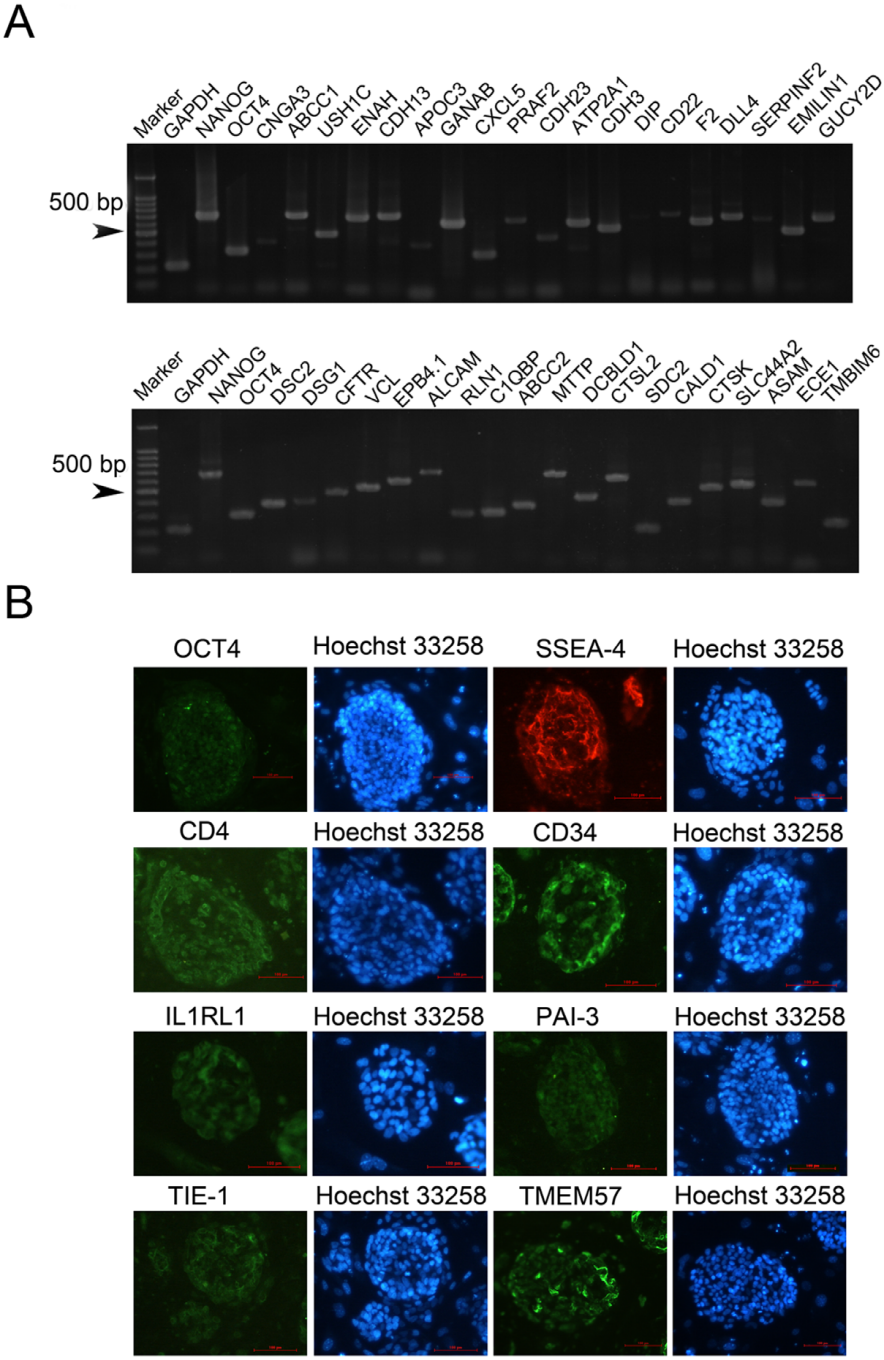
hES cells expressed tissue specific cell surface proteins. A. RT-PCR analysis demonstrated that hES cells expressed tissue specific cell surface proteins. B. Immunocytochemistry staining showed that mES cells expressed CD4, CD34, Il1Rl1, PAI-3, TIE-1 and TMEM57 along with hES specific marker OCT4 and SSEA4.

As hES cells have been shown to express tissue-specific genes at low levels and as we have demonstrated that hES cells express the Aire gene, we speculated that hES cells also express a large repertoire of tissue-specific surface proteins. To this end, we evaluated the expression of the mRNA transcripts of a panel of tissue- and lineage-specific surface protein genes in hES cells by RT-PCR (Some of them were examplified in Figure 6A). Table 1 summarizes the list of tissue- and lineage-specific surface protein genes expressed in hES cells detected by RT-PCR. Our results indicate that like mES cells, hES cells also express a large repertoire of tissue- and lineage-specific genes. Interestingly, the panel of tissue- or lineage-specific genes expressed in the hES cells was markedly different from that of the mES cells. Immunofluorescent studies in hES cells confirmed the expression of several tissue- or lineage-specific proteins, including CD4 (T helper cells), CD34 (hematopoietic cells), IL1RL1 (leukocyte), PAI-3 (liver), TIE-1, and TMEM57 (Figure 6B). Importantly, immunofluorescent analysis of hES cells also detected both OCT4 and SSEA-4, two hES cell markers [33], verifying the undifferentiated status of the hES cells utilized in this study. These results indicated that hES cells also globally expressed tissue-specific surface proteins.

**Table 1:** Tissue specific cell surface proteins expressed on hES cells.

Tissue	TRA
Cardiovascular	CDH13;CDH3;DLL4; BSG
Endothelia	Amot; CD31; KDR; Tie-1;
Epithelia	DCD;EVPL;EPN3;CFTR;DSC2;DSG1;ENAH;LIMA1;SDC4;ENAM;SLC44A2;ABCA4;CDH23; CNGA3
Gastrointestinal	CA9;APOA1;APOC3;ABCC2;USH1C;PRAF2;MTTP;PTGER3
Hematopoietic &immune	ALCAM; CD4;CD22; CD34;EPB41;CD79B; FCER1G;NCKAP1L;
Kidney	BBS1;CDH16;SLC12A1
Liver	AGXT;SERPINF2;ABCB11;F2;C1QBP;HPX;PAI-3
Muscle	ATP2A1;CACNB1;CALD1;CROT;DYSF;UTRN;VCL
Neural	HTR1D;CEND1;CHRM2;CNTN4;GABRA5;GRIA4; TMEM57
Reproductive	GANAB;AMIGO2;RLN1;CTSL2;CXCL5;CXCL6;CMTM1;TMBIM6;DNAJB13
Respiratory	ABCC1;BMPER;DIP;DCBLD1;EMILIN1;EMCN;ECE1

## Discussion

The proper activation of pluripotency-associated genes like Oct4 and Nanog and the suppression of differentiation-regulating genes like the Hox genes were thought to be major mechanisms for the maintenance of pluripotency in ES cells [4], [34]. However, recent studies have shown that promoters of most genes in the genome of human ES cells were marked with the activating epigenetic modification H3K4meth3 [10]. Most of the suppressed differentiation-regulating transcription factors were marked with the bivalent domain, which consisted of both activating H3K4meth3 and suppressing H3K27meth3 [35]. The mRNA transcripts of most genes, even those not modified with either activating or suppressing epigenetic modifications, could be detected in ES cells [9], [36]. Therefore, most genes in the ES cells were set in a transcriptionally poised state, where the mRNAs were produced only occasionally. An important issue to address is whether the global gene expression phenomenon is just leaky gene expression resulting from the elastic and dynamic chromatin structure of ES cells or whether it has functional significance. Here, we report that at least for cell-surface proteins, a large variety of globally expressed genes were translated into proteins. The accessibility of these surface proteins from the extracellular space indicated that many of them had been presented in their mature functional form rather as precursors that remain in the ER and Golgi apparatus. As calculated from the detection limit of mass spectrometry and the amount of cells used in this study, the least abundant surface protein identified here should be presented on each cell at the level of hundreds of copies [37]. This number is sufficient for the functional performance of the proteins. These evidences support the argument that global gene expression is functional in ES cells.

### ES cells are versatile signal transformers

In contrast to terminally differentiated cell types, which mount restricted responses to various stimuli, ES cells mount essentially infinite responses, differentiating to all cell types in the organism. Two seemingly contradictory responses of ES cells to stimulus were observed. First, ES cells respond differently to different stimuli, to the same stimulus of different strengths and to different combinations of stimuli. For example, Zansdtra et al. showed a concentration-dependent effect of LIF on mES cells [38]. Second, ES cells respond differently to the same stimulus. For example, when cultured in suspension, ES cells in identical medium form embryonic bodies, which consist of cells of the three germ layers. The evidence we present here that ES cells globally express a large diversity of surface signaling molecules in a heterogeneous manner may partially explain these properties. The versatility of signaling receptors could enable ES cells to transform diverse stimulus into highly variable differentiation behaviors. In addition, the heterogeneity of the signal-accepting ability caused by the differential expression of signaling molecules could enable ES cells to transform the same stimulus to different differentiation behaviors. The global and heterogeneous expression of signaling molecules make ES cells versatile signal transformers, ensuring their plasticity and pluripotency.

### Implications of the population heterogeneity of ES cells

It has been demonstrated that ES cells heterogeneously express genes like Nanog, Rex-1, Stella and CD133 [26], [27], [28], [39]. The different subpopulations sorted according to these markers possess different self-renewal abilities and differentiation potentials. Moreover, a recent study has shown that a subpopulation of undifferentiated mES cells that express the primitive endoderm (PrEn)-specific gene Hex at very low level has early PrEn properties and that their differentiation into PrEn was favored [40]. Our results showed that undifferentiated mES cells actually expressed a large repertoire of tissue-specific surface proteins at low levels and the expression of many of them tended to be heterogeneous. These results indicate that ES cells actually consist of different subpopulations expressing different tissue-specific proteins and have different differentiation tendencies. Our results supported the idea that ES cells are a equilibrium population that consist of subpopulations of different differentiation potentials.

### Implications for surface marker screening of ES cells

ES cell surface markers are valuable tools for the characterization, quality control and purification of ES cells. Extensive efforts have been mounted for decades to discover the surface markers of ES cells. Until now, the most widely used specific ES cell surface markers (SSEA1, SSEA3, SSEA4, Tra-1-60, Tra-1-81) were all glycan epitopes on glycoproteins or glycolipids [33], [41]. Although some proteins like CD9, HSPA8 and PODXL have been proposed to be specific surface markers of ES cells, they are all only relatively specific and only expressed in certain tissues, according to data from the human protein atlas [16], [33], [42], [43], [44]. The question remains whether or not there is a specific surface protein marker that is exclusive to ES cells. Our results showed that mES cells globally expressed a large repertoire of tissue-specific cell surface proteins. It indicated that, at least for mES cells, an exclusive, specific surface protein marker is not easy to identify. The same thing may also hold true for hES cells because we have shown that hES cells express tissue-specific surface proteins. Therefore, new strategies should be employed to screen for surface markers of ES cells. Hematopoietic stem cells (HSC) are another type of stem cells that exhibit promiscuous surface protein expression [45]. HSCs express genes specific to differentiated hematopoietic cells and other cell types, like neural cells. Researchers have determined quantitatively the combination of markers, like the lineage markers Sca-1, c-Kit or SLAMs, to identify HSCs [46], [47], [48], [49]. We suggest that the same idea is applicable to ES cells. The quantitative combination of a group of surface proteins of different tissue specificities could identify an ES-specific surface protein pattern. Therefore, perhaps efforts should be shifted from screening for exclusive, specific markers to determining the combinations.

In conclusion, we demonstrated that mES cells globally expressed cell surface proteins of diverse functions and tissue specificities. Our results support the idea that global gene expression in ES cells is functional. Moreover, our results indicate that ES cells are versatile in their signal reception and transduction ability. Our results also have profound implications for understanding the functional and population properties of ES cells and they also indicate new strategies for surface marker screening.

## Materials and Methods

### Ethics Statement

We do not require a Ethics Statement because we only used mouse cell lines and commercially available human cell lines. No animals or human samples were used. And the approval of a named review board institution or ethics committee is not needed for the same reason.

### Cell lines and Cell culture

Gamma irradiation inactivated mouse embryonic fibroblast (MEF) feeder cells isolated from the embryos of ICR mice at gestational day 13.5 were purchased from Invitogen (Invitrogen, Carlsbad, CA). MEFs were thawed in DMEM supplemented with 10% fetal bovine serum(Invitrogen) at 37°C and plated at a density of 4×10^4^ cells/cm^2^ for ES culture.

Mouse ES(mES) cell D3 line was purchased from ATCC (Manassas, VA). ZJ1 and ZJ2 mouse ES cell lines were established in our laboratory[50]. mES cells (D3, ZJ1 and ZJ2) were cultured on gamma irradiation inactivated MEFs in DMEM supplemented with 15% fetal bovine serum (Invitrogen) and 1000 ng/ml LIF (Millipore, Billerica, MA) at 37°C in 5% CO_2_. The pluripotency of the mES cells was routinely analyzed using ALP staining Kit(Sigma), SSEA-1 staining and teratoma formation. In addition, the karyotype was checked routinely.

Human embryonic stem cells H9 were purchased from WiCell (Madison,WI) and cultured on gamma irradiation inactivated MEFs in Knockout DMEM supplemented with 20% KOSR (Invitrogen) and 1000 ng/ml bFGF (Millipore) at 37°C in 5% CO_2_. The pluripotency of the hES cells was routinely analyzed using ALP staining (Sigma), SSEA-4 staining and teratoma formation. In addition, the karyotype was checked routinely.

### Cell surface labeling and affinity purification

The mES D3 cells (5×10^8^) cultured gamma irradiation inactivated MEFs were trypsinized to single cells and plated on gelatin-coated 100 mm culture dishes. After 1 hour, most MEFs adhered to the culture dish. The mES cells in suspension were collected and biotin labeled.

For biotin labeling, the cells were incubated with 1 mg/ml Sulfo-NHS-SS-Biotin (Pierce, Rockford, IL) in PBS for 30 minutes. Excess biotin was quenched using 10 mM Glycin for 10 minutes, and then the cells were washed three times with PBS. Next, the cells were homogenized in ice-cold cell lysis buffer (50 mM Tris-Cl, pH 7.4, 1% NP-40 substitute (Sigma), 150 mM NaCl, 1 mM EDTA, 1 mM PMSF(Sigma)) using a Dounce Homogenizer (30 strokes). The homogenate was put on ice for 1 hour with gentle vortexing to extract the membrane proteins. After that, the homogenate was centrifuged at 12000 g for ten minutes to remove the nuclei, unbroken cells and cell fragments. The supernatant was mixed with streptavidin-coupled LATEX (300 nm diameter) beads and vortexed at 4°C for 1 hour. The LATEX beads were precipitated by centrifugation and washed twice with 0.1 M Na_2_CO_2_ and once with 1 M KCl to remove the contaminant proteins. After that, the disulfide bonds linking biotin and the purified proteins were cleaved by 100 mM DTT(Sigma) to elute the purified proteins. Approximately 100 µg of membrane protein could be purified from 10^8^ cells. The labeling efficiency was monitored using FITC-streptavidin staining.

### SDS-PAGE separation

The purified proteins were separated by SDS-PAGE using a 12.5% SDS-PAGE gel. After electrophoresis, the gels were stained with Coomassie Blue R250(Sigma) and then dissected into 8 bands for LC-MS/MS analysis.

### Enzyme digestion, LC-MS/MS analysis and database searching

The enzyme digestion was performed as previously described [51]. The peptides from each band were separated on a Paradigm MS4N Nano/Capillary HS MDLC (Michrom Bioresources, Inc. USA) using a 100 µm×150 mm C18 reverse phase column. The LC separation was conducted with a linear gradient of 5–35% buffer B for 50 min, followed by 35–90% buffer B for 10 minutes, followed by 90% buffer B for 10 minutes (buffer A: 0.1% formic acid in a 2% acetonitrile H_2_O solution; buffer B: 0.1% formic acid in a 98% acetonitrile H2O solution) at a flow rate of 500 nl/min. The separated peptides were then analyzed on a LTQ-MS (Thermol, USA) coupled with a Michrome Advanced nanospray apparatus (Microm). The peak list files generated by the Bioworks software (Applied Biosystems, USA) using the default parameters were searched against databases for protein identification using the Sequest software. The searching parameters were: for 2 or 3 valent ions, Xcorr ≥2; for 1 valent ion, Xcorr ≥1.5;,Deltacn ≥0.1; and two nonredundant peptides identified on a unique protein.

### Antibodies and immunocytochemistry

The following antibodies were used: Oct-4 (R&D MAB 1759), SSEA-1 (R&D MAB2155), CD34 (HUABIO, Hangzhou, China), c-Kit (HUABIO), EGFR (HUABIO), BMPR2 (HUABIO), E-Cadherin (HUABIO 0407-25), BMP2 (HUABIO 0806-2), GM-CSF Rα (HUABIO 0804-8), CD4 (HUABIO), TIE-1 (HUABIO 0804-11), PAI-3 (HUABIO), TMEM57(HUABIO), R-PE-conjugated goat anti-rabbit IgG (Proteintech Chicago, USA), and Alexa 488-conjugated goat anti-rabbit IgG (Invitrogen).

For double staining, the cells were fixed using 4% paraformaldehyde according to the standard protocol, blocked with blocking/permeating buffer (PBS with 10% goat serum and 0.3% Triton-X100) and then incubated with Rat anti-human Oct4 monoclonal antibody overnight at 4°C. After wash, the cells were incubated with a Alexa 488-conjugated Goat-anti Rat for 1 hour at 37°C. After wash, the cells were incubated with rabbit polyclonal antibodies against cell surface molecules for 1 hour at 37°C. After wash, the cells were Alexa 555-conjugated Goat-anti Rabbit for 1 hour at 37°C and then observed under a LSM500 Confocal Microscope (Zeiss, Germany). For single staining, the cells were fixed using 4% paraformaldehyde according to the standard protocol, blocked with blocking/permeating buffer (PBS with 10% goat serum and 0.3% Triton-X100) and then incubated with primary antibodies for 1 hour at 37°C. After washing, the cells were incubated with a Alexa 488-conjugated secondary antibodies for 1 hour at 37°C and then observed under a fluorescent microscope(Nikon, Japan).

Biotin-labeled mES cells were fixed with 4% paraformaldehyde overnight at 4°C and then stained with FITC-conjugated streptavidin (Sigma) for 30 minutes to monitor the surface labeling.

### RT-PCR

RT-PCR was performed as previously described [12]. Total RNA was extracted using the Trizol Reagent (Takara, Japan), retro-transcribed and then PCR-amplified. The primers were designed using the PRIMER PREMIER 5 software.

### ALP staining

ALP staining was performed with an ALP assay kit (Sigma).

### Flow cytometry

The mES cells were dissociated with 0.05 mM EDTA and then washed with PBS and 3% FBS to remove the EDTA. The cells were then incubated with a primary antibody for 1 hour on ice. After thorough washes, the cells were incubated with fluorescent secondary antibodies for 30 minutes on ice. The cells were then washed with PBS and analyzed by BDLSR flow cytometry (BD Biosciences, San Jose, CA).

### Western blotting

Western blotting was carried out as described previously[52]. Briefly, ES cells were harvested in lysis buffer (50 mM Tris-HCl pH 7.4, 1% NP-40, 1% SDS), and equal amounts of the protein lysate were separated by electrophoresis on a 12.5% Laemmli SDS-polyacrylamide gel. The proteins were then transferred onto PVDF membranes. After incubation with primary and secondary antibodies, the membranes were developed using an ECL kit purchased from GE Healthcare Life Sciences (Piscataway, NJ).

### Bioinformatics analysis

The subcellular localization of the proteins was annotated according to the Swissprot annotation, the Sosui prediction software and the literature. Proteins containing transmembrane domains, secreted proteins and proteins annotated as cell surface proteins by either Swissprot or the existing literature were all considered cell surface proteins. A gene ontology (GO) analysis was done using the DAVID software and database [30], [31]. The molecular pathways were analyzed according to the KEGG pathway annotations. The tissue specificity of the surface proteins was annotated according to Uniprot annotations.

## Supporting Information

Table S1 **The list of cell surface proteins identified in this study.**
(DOC)
