# Supplementary material for: Global Expression of Cell Surface Proteins in Embryonic Stem Cells
Source: PLoS One. 2010 Dec 29;5(12):e15795. doi: 10.1371/journal.pone.0015795 (PMC3012103; doi:10.1371/journal.pone.0015795)
Supplement: Table S1 — The list of cell surface proteins identified in this study. (DOC) [file pone.0015795.s001.doc]

Cell Surface proteins identified on mES cells.

| protein name | NCBI Gi |
| --- | --- |
| 2,4-dienoyl CoA reductase 1, mitochondrial | 13385680 |
| 3-hydroxy-3-methylglutaryl-Coenzyme A reductase | 160358778 |
| 3-hydroxy-3-methylglutaryl-Coenzyme A synthase 1 | 31981842 |
| 3-hydroxybutyrate dehydrogenase (heart, mitochondrial) | 170014720 |
| 3-hydroxyisobutyryl-Coenzyme A hydrolase | 22122625 |
| 5' nucleotidase, ecto | 6754900 |
| 5,10-methylenetetrahydrofolate reductase | 31543271 |
| a disintegrin and metallopeptidase domain 23 preproprotein | 6752968 |
| disintegrin and metalloproteinase domain-containing protein 10 precursor | 150378458 |
| a disintegrin and metalloprotease domain 19 (meltrin beta) | 6752966 |
| a disintegrin and metalloprotease domain 4b | 111955131 |
| a disintegrin and metalloproteinase domain 6-like | 57222276 |
| aarF domain containing kinase 2 | 30725855 |
| aarF domain containing kinase 5 | 169234776 |
| acetylcholinesterase | 13928664 |
| acetyl-Coenzyme A acyltransferase 1 | 18700004 |
| acetyl-Coenzyme A acyltransferase 2 | 29126205 |
| acetyl-Coenzyme A carboxylase alpha | 125656173 |
| achalasia, adrenocortical insufficiency, alacrimia | 241982696 |
| acid phosphatase 2, lysosomal | 29150253 |
| acid phosphatase, prostate isoform 1 | 46575782 |
| activated leukocyte cell adhesion molecule | 31791059 |
| activin receptor IIB | 6680634 |
| acyl-Coenzyme A dehydrogenase, very long chain | 23956084 |
| AD158 | 19923058 |
| adenosine monophosphate deaminase 2 (isoform L) | 21311925 |
| adenylate cyclase 4 | 17978250 |
| adenylate cyclase 8 | 124430532 |
| adenylate cyclase 9 | 150378487 |
| adipsin | 7304867 |
| ADP-ribosylation factor-like 6 interacting protein 1 | 45433590 |
| afamin | 125347464 |
| agrin | 42490751 |
| A-kinase anchor protein 7 | 145864493 |
| serine--pyruvate aminotransferase, mitochondrial | 111038130 |
| alcohol dehydrogenase 4 (class II), pi polypeptide | 121247379 |
| aldehyde dehydrogenase 16 family, member A1 | 26080429 |
| alkaline phosphatase, tissue-nonspecific isozyme precursor | 160333226 |
| alpha glucosidase 2 alpha neutral subunit | 6679891 |
| amiloride-sensitive cation channel 1, neuronal (degenerin) iso1 | 6680622 |
| amphoterin induced gene and ORF | 51988881 |
| anaplastic lymphoma kinase | 110347475 |
| androgen-induced 1 | 13384848 |
| angiomotin | 125346196 |
| angiomotin like 2 | 119120838 |
| angiopoietin 1 | 46048213 |
| angiopoietin-like 2 | 31560520 |
| angiopoietin-like 3 | 33469117 |
| annexin A3 | 160707925 |
| anti-Mullerian hormone | 85861194 |
| apolipoprotein E | 163644329 |
| aquaporin 1 | 6680710 |
| argininosuccinate synthetase | 6996911 |
| aspartyl aminopeptidase | 161016820 |
| aspartyl beta-hydroxylase isoform 1 | 125628659 |
| astrotactin 1 | 46488926 |
| ATP citrate lyase | 29293809 |
| ATP synthase, H+ transporting mitochondrial F1 complex, beta | 31980648 |
| ATP synthase, H+ transporting, mitochondrial F1 complex, alpha | 6680748 |
| ATP synthase, H+ transporting, mitochondrial F1 complex, gamma | 163838641 |
| ATPase type 13A1 | 283135194 |
| ATPase type 13A5 | 257196258 |
| ATPase, Ca++ transporting, cardiac muscle, slow twitch 2 | 6806903 |
| ATPase, Ca++ transporting, fast twitch 1 | 36031132 |
| ATPase, Ca++ transporting, ubiquitous | 31542159 |
| Atpase, class VI, type 11C isoform a | 83745137 |
| ATPase, H+ transporting, lysosomal V0 subunit a isoform 2 | 83627707 |
| ATPase, H+/K+ transporting, nongastric, alpha polypeptide | 157168326 |
| ATP-binding cassette 1, sub-family A, member 1 | 90568038 |
| ATP-binding cassette transporter sub-family A member 9 | 153792543 |
| ATP-binding cassette, sub-family A (ABC1), member 6 | 262118230 |
| ATP-binding cassette, sub-family A, member 7 | 15451840 |
| ATP-binding cassette, sub-family B (MDR/TAP), member 4 | 161086924 |
| ATP-binding cassette, sub-family B, member 10 | 9506367 |
| bile salt export pump | 120432047 |
| ATP-binding cassette, sub-family C (CFTR/MRP), member 12 | 52138554 |
| ATP-binding cassette, sub-family C, member 2 | 116063566 |
| multidrug resistance-associated protein 6 | 145275191 |
| ATP-binding cassette, sub-family D, member 3 | 60218877 |
| ATP-binding cassette, sub-family D, member 4 | 226052788 |
| ATP-binding cassette, subfamily E, member 1 | 114205431 |
| ATP-binding cassette, sub-family F (GCN20), member 1 | 39930335 |
| AXL receptor tyrosine kinase | 31542164 |
| basal cell adhesion molecule | 10048460 |
| basigin | 34915988 |
| Bcl2-like 10 | 7304927 |
| BCS1-like | 21313544 |
| beta 1,4-N-acetylgalactosaminyltransferase-transferase-III | 38566700 |
| beta1,4-N-acetylgalactosaminyltransferase IV | 58372116 |
| beta-site APP-cleaving enzyme 2 | 244798416 |
| blood vessel epicardial substance | 13195598 |
| bone morphogenetic protein 1 | 42734447 |
| bone morphogenetic protein 15 | 7106259 |
| bone morphogenic protein receptor, type II | 6680804 |
| brain-selective kinase 2 isoform gamma | 58036485 |
| brain-specific angiogenesis inhibitor 1 | 92110033 |
| bromodomain and WD repeat domain containing 2 | 227908800 |
| butyrate-induced transcript 1 | 171184435 |
| Ca<2+>dependent activator protein for secretion | 70906474 |
| cadherin 1 | 6753374 |
| cadherin 11 | 114687888 |
| cadherin 12 | 56606025 |
| cadherin 3 isoform b | 45496816 |
| cadherin 4 | 6753376 |
| cadherin 6 | 110347562 |
| cadherin 7, type 2 | 27370290 |
| cadherin EGF LAG seven-pass G-type receptor 1 | 115648153 |
| cadherin EGF LAG seven-pass G-type receptor 2 | 114050895 |
| calcium channel, voltage-dependent, beta 3 subunit | 113680271 |
| calcium channel, voltage-dependent, N type, alpha 1B subunit | 110225366 |
| calcium channel, voltage-dependent, P/Q type, alpha 1A subunit | 117968623 |
| calcium channel, voltage-dependent, R type, alpha 1E subunit | 117968623 |
| calmegin | 86262138 |
| calnexin | 6671664 |
| cannabinoid receptor 1 (brain) | 6724315 |
| carbohydrate sulfotransferase 10 | 70908368 |
| carboxypeptidase A5 | 21362335 |
| carboxypeptidase N, polypeptide 2 homolog | 147904569 |
| probable carboxypeptidase X1 precursor | 228008326 |
| carboxypeptidase Z | 120407066 |
| cartilage acidic protein 1 | 110626040 |
| catechol-O-methyltransferase domain containing 1 | 33468999 |
| cation channel, sperm associated 3 | 30350218 |
| CD109 antigen | 23346525 |
| CD180 antigen | 117320545 |
| CD209b antigen isoform a | 83699398 |
| CD209c antigen | 18777733 |
| CD2-associated protein | 125987599 |
| T-cell surface glycoprotein CD3 delta chain precursor | 227498961 |
| CD5 antigen-like | 160358823 |
| CD7 antigen | 6806905 |
| T-cell surface protein tactile precursor | 160333634 |
| CDP-diacylglycerol--inositol 3-phosphatidyltransferase | 21362277 |
| CDW92 antigen | 227499980 |
| CEA-related cell adhesion molecule 14 | 13385448 |
| cell adhesion molecule with homology to L1CAM | 110347545 |
| cell adhesion molecule-related/down-regulated by oncogenes | 160333799 |
| cerebellin 4 precursor | 28274690 |
| chemokine (C-C motif) ligand 28 | 9931984 |
| chemokine-like factor superfamily 6 | 21313082 |
| chloride channel 6 | 6753434 |
| chloride channel calcium activated 1 | 32964827 |
| chloride channel calcium activated 3 | 8567336 |
| chloride channel CLIC-like 1 | 21704070 |
| chloride intracellular channel 1 | 15617203 |
| cholecystokinin | 13624301 |
| cholinergic receptor, nicotinic, alpha polypeptide 5 | 46559780 |
| cholinergic receptor, nicotinic, beta polypeptide 3 isoform 2 | 27532978 |
| cholinergic receptor, nicotinic, epsilon polypeptide | 6752950 |
| cholinergic receptor, nicotinic, gamma polypeptide | 119964696 |
| chondroitin sulfate proteoglycan 4 | 146231960 |
| chondroitin sulfate proteoglycan 6 | 36031035 |
| CLST 11240 protein | 18252786 |
| coagulation factor VIII | 238624180 |
| coagulation factor X | 110625994 |
| collagen 28 | 83745131 |
| collagenous repeat-containing | 13562098 |
| collectin-10 precursor | 226053018 |
| complement component 1, q subcomponent binding protein | 112181167 |
| complement component 3 | 126518317 |
| complement component 8, beta subunit | 33563297 |
| complement component 9 | 15375312 |
| complement factor H-related protein | 237757318 |
| contactin 1 | 6680954 |
| contactin 6 | 31980846 |
| contactin associated protein-like 2 isoform a | 165377150 |
| corticotropin releasing hormone | 45429990 |
| COX18 cytochrome c oxidase assembly homolog | 254540218 |
| coxsackievirus and adenovirus receptor isoform b | 6857775 |
| C-type lectin domain family 14, member a | 31541811 |
| cyclic nucleotide gated channel alpha 1 | 112181173 |
| cysteine rich transmembrane BMP regulator 1 (chordin like) | 71895029 |
| cytochrome b5 reductase 1 (B5R.1) | 21312524 |
| cytochrome c oxidase subunit 8C | 84794657 |
| cytochrome c-1 | 13385006 |
| cytochrome P450, family 19, subfamily a, polypeptide 1 | 6681099 |
| cytochrome P450, family 2, subfamily c, polypeptide 39 | 242332605 |
| cytochrome P450, family 2, subfamily s, polypeptide 1 | 21311915 |
| cytochrome P450, family 3, subfamily a, polypeptide 13 | 6681115 |
| cytochrome P450, family 4, subfamily x, polypeptide 1 | 51592065 |
| cytokine induced protein 29 kDa | 13384730 |
| cytokine receptor-like factor 1 | 9055196 |
| cytoskeleton-associated protein 4 | 62526118 |
| defender against cell death 1 | 6753598 |
| defender against cell death 1 | 6753598 |
| degenerative spermatocyte homolog 1 | 6681175 |
| deleted in colorectal carcinoma | 117168295 |
| Der1-like domain family, member 1 | 13195638 |
| desmoglein 2 | 161016843 |
| desmoglein 3 | 110625833 |
| diacylglycerol O-acyltransferase 2 | 16975490 |
| diacylglycerol O-acyltransferase 2 | 16975490 |
| diffuse panbronchiolitis critical region 1 | 84370308 |
| dipeptidylpeptidase 4 | 6753674 |
| dipeptidylpeptidase 9 | 255003757 |
| dolichyl-di-phosphooligosaccharide-protein glycotransferase | 46195798 |
| dystroglycan 1 | 33859532 |
| dystroglycan 1 | 33859532 |
| ectonucleotide pyrophosphatase/phosphodiesterase 6 | 28893409 |
| elastin microfibril interfacer 1 | 19527130 |
| elastin microfibril interfacer 3 | 37620147 |
| Ellis van Creveld syndrome 2 homolog | 22122329 |
| endoglin | 226437647 |
| lysophosphatidic acid receptor 1 | 171543831 |
| endothelial differentiation, sphingolipid G-protein-coupled receptor | 45433562 |
| angiopoietin-1 receptor | 111185951 |
| endothelin converting enzyme 2 | 153945738 |
| Eph receptor A2 | 32484983 |
| Eph receptor A3 | 31982448 |
| Eph receptor A4 | 34328113 |
| Eph receptor A6 | 145312274 |
| Eph receptor A8 | 154240697 |
| Eph receptor B3 | 33859548 |
| ephrin B1 | 6753726 |
| erythrocyte protein band 4.2 | 7305035 |
| erythrocyte protein band 7.2 | 7710018 |
| augurin precursor | 228008374 |
| Ewing sarcoma breakpoint region 1 | 88853581 |
| exostoses (multiple)-like 3 | 46852189 |
| exostosin 1 | 112807209 |
| extracellular matrix protein 2 | 59858563 |
| F11 receptor | 27734847 |
| fibronectin type III domain-containing protein 3B | 170932548 |
| Fanconi anemia, complementation group D2 | 282721083 |
| fatty acid synthase | 93102409 |
| FERM domain containing 3 | 27370318 |
| fibrillin 1 | 118197277 |
| fibrillin 2 | 118136302 |
| fibroblast growth factor 12 isoform a | 34098966 |
| fibroblast growth factor 23 | 12083589 |
| fibroblast growth factor 8 | 22094093 |
| fibroblast growth factor binding protein 3 | 139948267 |
| fibronectin 1 | 46849812 |
| fibronectin leucine rich transmembrane protein 3 | 30410006 |
| flavin containing monooxygenase 1 | 6753890 |
| flavin containing monooxygenase 4 | 21450117 |
| flotillin 2 isoform 2 | 6679811 |
| FMS-like tyrosine kinase 3 | 122937353 |
| follistatin-like 4 | 57527848 |
| Fras1 related extracellular matrix protein 1 | 52138540 |
| Fras1 related extracellular matrix protein 2 | 71051607 |
| frizzled 1 | 31981157 |
| frizzled 5 | 47059488 |
| fucosyltransferase 10 isoform Fut10A | 60302885 |
| fucosyltransferase 7 | 7305073 |
| G protein-coupled receptor 109B | 13507640 |
| G protein-coupled receptor 111 | 134949047 |
| G protein-coupled receptor 153 | 33859807 |
| G protein-coupled receptor 153 | 33859807 |
| G protein-coupled receptor 162 | 7305103 |
| G protein-coupled receptor 35 | 157267234 |
| G protein-coupled receptor 37 | 160298211 |
| G protein-coupled receptor 55 | 111185938 |
| G protein-coupled receptor 82 | 28316782 |
| G protein-coupled receptor 84 | 13507672 |
| extracellular calcium-sensing receptor precursor | 148762954 |
| G protein-coupled receptor, family C, group 6, member A | 23346475 |
| G protein-regulated inducer of neurite outgrowth 1 | 84000009 |
| galactose-3-O-sulfotransferase 1 | 31980855 |
| galactose-3-O-sulfotransferase 2 | 224922779 |
| gamma-aminobutyric acid (GABA-A) receptor, subunit alpha 1 | 6753936 |
| gamma-aminobutyric acid (GABA-A) receptor, subunit alpha 3 | 247269171 |
| gamma-aminobutyric acid (GABA-A) receptor, subunit gamma 2 iso | 83921591 |
| gamma-aminobutyric acid A receptor, gamma 1 precursor | 31542878 |
| gamma-glutamyltransferase 6 precursor | 117647222 |
| gamma-glutamyltransferase-like activity 1 | 31542891 |
| ganglioside-induced differentiation-associated protein 1-like 1 | 160420342 |
| gap junction membrane channel protein beta 3 | 6680013 |
| gap junction membrane channel protein beta 4 | 6680015 |
| gene trap locus 3 | 225543576 |
| glucosaminyl (N-acetyl) transferase 2 isoform B | 39995102 |
| glucosidase 1 | 31981106 |
| glutamate receptor, ionotropic, delta 2 | 6680091 |
| glutamate receptor, ionotropic, kainate 1 isoform a | 84000015 |
| glutamate receptor, ionotropic, kainate 5 (gamma 2) | 160298215 |
| glutamate receptor, ionotropic, NMDA2C (epsilon 3) | 75709202 |
| glutamate receptor, ionotropic, NMDA2D (epsilon 4) | 144922606 |
| glutamate receptor, ionotropic, N-methyl D-aspartate-like 1A | 30519919 |
| glutamate receptor, metabotropic 3 | 32469489 |
| glycine receptor, alpha 1 subunit | 31982694 |
| glycine receptor, alpha 2 subunit | 34556195 |
| glycoprotein A33 (transmembrane) | 11037800 |
| glycoprotein m6a | 23957686 |
| glycoprotein, synaptic 2 | 19923070 |
| golgi apparatus protein 1 | 6677905 |
| golgi autoantigen, golgin subfamily a, 5 | 7305095 |
| golgi phosphoprotein 4 | 30424814 |
| golgi transport 1 homolog B | 13385354 |
| GPI-anchored membrane protein 1 | 42558248 |
| G-protein coupled receptor 119 | 32306530 |
| growth differentiation factor 1 | 51036615 |
| growth differentiation factor 7 | 33859664 |
| growth hormone receptor | 114520600 |
| GTPase, IMAP family member 1 | 28559037 |
| heat shock 40kD protein 2 | 10181196 |
| heat shock 70kD protein 5 (glucose-regulated protein) | 254540166 |
| heat shock 70kDa protein 14 isoform 1 | 82880662 |
| heat shock 70kDa protein 4 like | 40254361 |
| heat shock factor 2 | 226530977 |
| heat shock protein 1 (chaperonin) | 183396771 |
| heat shock protein 1, alpha | 6754254 |
| heat shock protein 1, beta | 40556608 |
| heat shock protein 105 | 114145505 |
| heat shock protein 1-like | 124339838 |
| heat shock protein 2 | 50345978 |
| heat shock protein 4 | 112293266 |
| heat shock protein 9A | 162461907 |
| Hedgehog-interacting protein | 160358774 |
| hemopexin | 160358829 |
| heparanase | 22779887 |
| heterogenous nuclear ribonucleoprotein U | 160333923 |
| histamine N-methyltransferase | 17978276 |
| histocompatibility 2, M region locus 10.4 | 29244030 |
| hyaluronan mediated motility receptor | 226693396 |
| hyaluronidase 3 | 254939507 |
| corticosteroid 11-beta-dehydrogenase isozyme 2 | 133778913 |
| potassium/sodium hyperpolarization-activated cyclic nucleotide-gated channel 3 | 6680191 |
| family with sequence similarity 83, member H | 269914118 |
| malectin precursor | 188497650 |
| hypothetical protein LOC209743 | 23943816 |
| hypothetical protein LOC210417 | 40254219 |
| hypothetical protein LOC210673 | 66793406 |
| hypothetical protein LOC212073 | 27369682 |
| hypothetical protein LOC215015 | 21703824 |
| hypothetical protein LOC215015 | 21703824 |
| coiled-coil domain-containing protein 109A | 168823441 |
| hypothetical protein LOC216820 | 21703854 |
| hypothetical protein LOC216881 | 31560748 |
| hypothetical protein LOC224171 | 125858491 |
| hypothetical protein LOC227612 | 31981884 |
| hypothetical protein LOC228684 | 85701796 |
| hypothetical protein LOC229588 | 67625729 |
| hypothetical protein LOC229722 | 85701810 |
| hypothetical protein LOC231225 | 71143116 |
| hypothetical protein LOC235300 | 85702201 |
| hypothetical protein LOC235461 | 118403316 |
| leucine-rich repeat and immunoglobulin-like domain-containing nogo receptor-interacting protein 3 | 269973933 |
| hypothetical protein LOC239463 | 169646258 |
| hypothetical protein LOC239691 | 268838809 |
| hypothetical protein LOC240334 | 27370248 |
| hypothetical protein LOC243274 | 27370350 |
| hypothetical protein LOC243407 | 206597524 |
| hypothetical protein LOC268706 | 30520193 |
| hypothetical protein LOC270084 | 27370522 |
| cation channel sperm-associated protein subunit beta | 257900479 |
| hypothetical protein LOC276852 | 31982246 |
| hypothetical protein LOC319719 | 171184441 |
| hypothetical protein LOC320271 | 86198339 |
| furry homolog-like isoform 2 | 119964714 |
| hypothetical protein LOC320487 | 38505263 |
| hypothetical protein LOC320609 isoform a | 83627727 |
| hypothetical protein LOC320709 | 30520273 |
| hypothetical protein LOC328370 | 87299621 |
| hypothetical protein LOC329659 | 29244450 |
| hypothetical protein LOC333564 | 56090556 |
| hypothetical protein LOC380702 | 85702193 |
| transmembrane protein 102 | 145966766 |
| hypothetical protein LOC381062 isoform 1 | 183074530 |
| hypothetical protein LOC381845 | 58037477 |
| hypothetical protein LOC382038 | 71274162 |
| inositol 1,4,5-triphosphate receptor-interacting protein precursor | 111185916 |
| hypothetical protein LOC433638 | 85702091 |
| hypothetical protein LOC442827 | 50582589 |
| coiled-coil domain-containing protein 69 | 115270981 |
| hypothetical protein LOC545279 | 85702258 |
| hypothetical protein LOC545893 | 85702266 |
| hypothetical protein LOC547349 | 68534959 |
| hypothetical protein LOC629756 | 87196519 |
| hypothetical protein LOC654818 | 112363105 |
| hypothetical protein LOC66087 | 28827824 |
| hypothetical protein LOC66270 isoform 2 | 31982730 |
| hypothetical protein LOC66272 | 13384872 |
| coiled-coil domain-containing protein 51 | 258679490 |
| hypothetical protein LOC66766 | 21313328 |
| coiled-coil domain-containing protein 47 precursor | 125628650 |
| hypothetical protein LOC67458 | 13385678 |
| hypothetical protein LOC68734 | 46849745 |
| hypothetical protein LOC68796 | 31559970 |
| hypothetical protein LOC68964 isoform a | 219689064 |
| hypothetical protein LOC69900 | 30519955 |
| hypothetical protein LOC70561 | 27501462 |
| hypothetical protein LOC70564 | 27229101 |
| hypothetical protein LOC70612 | 213972600 |
| hypothetical protein LOC71517 | 270047485 |
| hypothetical protein LOC71782 | 40789239 |
| hypothetical protein LOC71886 | 239937493 |
| hypothetical protein LOC71997 | 21312548 |
| hypothetical protein LOC72055 | 258645131 |
| hypothetical protein LOC72175 | 31541926 |
| hypothetical protein LOC72503 isoform 1 | 262118187 |
| tetratricopeptide repeat protein 39C | 257467486 |
| hypothetical protein LOC74315 | 262118218 |
| hypothetical protein LOC74319 | 239049442 |
| hypothetical protein LOC74442 | 27229211 |
| hypothetical protein LOC74653 | 21312828 |
| hypothetical protein LOC75698 | 122891876 |
| 2-C-methyl-D-erythritol 4-phosphate cytidylyltransferase-like protein | 157412261 |
| sphingomyelin phosphodiesterase 4 isoform 1 | 257196240 |
| hypothetical protein LOC98238 | 19527026 |
| Ia-associated invariant chain | 29789020 |
| immunoglobulin superfamily, member 1 isoform 3 | 262050642 |
| Indian hedgehog | 14149643 |
| inhibin beta-C | 6754354 |
| insulin receptor-related receptor | 160333073 |
| insulin-like 5 | 6754358 |
| insulin-like growth factor 1 isoform 2 | 34576545 |
| insulin-like growth factor 2 receptor | 133778978 |
| insulin-like growth factor 2, binding protein 1 | 6753518 |
| insulin-like growth factor 2, binding protein 3 | 225543383 |
| insulin-like growth factor binding protein 7 | 226958445 |
| integral membrane protein 2B | 6680502 |
| integrin alpha 1 | 153791389 |
| integrin alpha 11 | 225703035 |
| integrin alpha 2b | 159110663 |
| integrin alpha 5 | 225903442 |
| integrin alpha 6 | 31982236 |
| integrin alpha V | 154240716 |
| integrin beta 1 (fibronectin receptor beta) | 45504394 |
| integrin beta 4 isoform 1 | 110735426 |
| integrin beta 6 | 10946686 |
| integrin, alpha E, epithelial-associated isoform 2 | 27370456 |
| interferon (alpha and beta) receptor 1 | 160358843 |
| interferon alpha 6T | 46047411 |
| interferon induced transmembrane protein 5 | 33504579 |
| interleukin 1 receptor, type II | 6754330 |
| interleukin 1 receptor-like 1 isoform a | 71037395 |
| interleukin 11 | 6680393 |
| interleukin 12 receptor, beta 1 | 159110576 |
| interleukin 12 receptor, beta 2 | 6680401 |
| interleukin 12b | 6680397 |
| interleukin 13 receptor, alpha 1 | 40254373 |
| interleukin 16 | 52138550 |
| interleukin 17 | 6754324 |
| interleukin 19 | 57977321 |
| interleukin 2 receptor, beta chain | 6680427 |
| interleukin 20 | 10946716 |
| interleukin 27 | 21704110 |
| interleukin 3 | 31982791 |
| JP-45 protein | 21312578 |
| junctophilin 1 | 10181140 |
| junctophilin 2 | 10947010 |
| potassium voltage-gated channel subfamily S member 1 | 112821679 |
| killer cell lectin-like receptor subfamily A, member 20 | 21361214 |
| killer cell lectin-like receptor subfamily C, member 2 | 148886677 |
| killer cell lectin-like receptor, subfamily A, member 19 | 21361222 |
| kinase insert domain protein receptor | 27777648 |
| kinectin 1 | 144922638 |
| klotho | 121247379 |
| klotho beta | 13626032 |
| lactate dehydrogenase 1, A chain | 6754524 |
| ladinin | 31981555 |
| torsin-1A-interacting protein 1 isoform 2 | 229608944 |
| laminin B1 subunit 1 | 114326497 |
| laminin gamma 3 | 66392579 |
| laminin, alpha 1 | 117168301 |
| laminin, alpha 2 | 117647249 |
| laminin, alpha 4 | 148747408 |
| laminin, beta 2 | 31982223 |
| laminin, beta 3 | 113865981 |
| laminin, gamma 1 | 153791270 |
| latent transforming growth factor beta binding protein 3 | 124487415 |
| latrophilin 3 | 58037543 |
| lecithin-retinol acyltransferase | 12963753 |
| galectin-9 isoform 1 | 226531119 |
| leishmanolysin-like (metallopeptidase M8 family) | 27370232 |
| lens epithelium-derived growth factor | 19527168 |
| lens intrinsic membrane protein 2 | 29244136 |
| leucine zipper-EF-hand containing transmembrane protein 1 | 9789997 |
| leukemia inhibitory factor receptor | 7305235 |
| limb and neural patterns | 32441290 |
| limbic system-associated membrane protein | 30425330 |
| lipase, gastric | 13385836 |
| lipocalin 12 | 58037499 |
| lipocalin 13 | 23956342 |
| lipocalin 3 | 6754514 |
| lipocalin-interacting membrane receptor | 21312884 |
| lipopolysaccharide-binding protein | 113865991 |
| low density lipoprotein receptor-related protein 1 | 124494256 |
| low density lipoprotein receptor-related protein 4 | 224994223 |
| low density lipoprotein-related protein 1B | 153792247 |
| low-density lipoprotein receptor-related protein 10 precursor | 31981254 |
| lymphocyte antigen 74 | 112293275 |
| LYRIC | 31982233 |
| lysophosphatidic acid acyltransferase zeta | 30520301 |
| lysophosphatidylglycerol acyltransferase 1 | 26986567 |
| macrophage receptor with collagenous structure | 6754640 |
| macrophage stimulating 1 (hepatocyte growth factor-like) | 254675205 |
| macrophage stimulating 1 receptor | 254826769 |
| male sterility domain containing 2 | 68448551 |
| mannan-binding lectin serine protease 1 | 148226524 |
| mannose binding lectin (A) | 6754654 |
| mannose-6-phosphate receptor, cation dependent | 14916479 |
| mannose-P-dolichol utilization defect 1 | 31981340 |
| lysosomal alpha-mannosidase precursor | 113195690 |
| mannosidase, alpha, class 1A, member 1 | 6678788 |
| MAS-related GPR, member A1 | 254692948 |
| MAS-related GPR, member A8 | 46430528 |
| mast cell protease 2 | 6678838 |
| mast cell protease 4 | 114205406 |
| matrix metalloproteinase 11 | 6678894 |
| neutrophil collagenase precursor | 160333381 |
| MEGF10 protein | 50233828 |
| cell surface glycoprotein MUC18 precursor | 160333901 |
| melanoma inhibitory activity 3 | 124001582 |
| membrane bound C2 domain containing protein | 33859650 |
| membrane metallo endopeptidase | 31543255 |
| membrane protein, palmitoylated | 6678924 |
| MAGUK p55 subfamily member 5 | 9625023 |
| membrane-spanning 4-domains, subfamily A, member 7 isoform b | 71043948 |
| meprin 1 alpha | 31982199 |
| mesoderm specific transcript | 6678866 |
| met proto-oncogene | 146198696 |
| methyltransferase like 2 | 255683413 |
| methyltransferase like 4 | 74315949 |
| microfibrillar-associated protein 1 | 13385728 |
| mitochondrial Ca2+-dependent solute carrier | 31560754 |
| MON2 homolog | 253683420 |
| mucolipin 1 | 16716463 |
| myeloid-associated differentiation marker | 148271073 |
| Na+/K+ -ATPase alpha 1 subunit | 21450277 |
| Na+/K+ -ATPase alpha 3 subunit | 21450321 |
| Na+/K+ -ATPase beta 1 subunit | 6753138 |
| atrial natriuretic peptide receptor 1 precursor | 113930718 |
| N-deacetylase/N-sulfotransferase (heparin glucosaminyl) 4 | 157042776 |
| immunoglobulin superfamily DCC subclass member 4 precursor | 146231956 |
| nerve growth factor receptor (TNFR superfamily, member 16) | 70794803 |
| netrin 2-like | 6754904 |
| neural cell adhesion molecule 2 | 6754920 |
| sn1-specific diacylglycerol lipase alpha | 154091036 |
| neuregulin 3 | 6679128 |
| neurofascin | 35215309 |
| neuroligin 3 | 262118191 |
| neuronal pentraxin 2 | 7949098 |
| neuronal pentraxin receptor | 36054013 |
| neuropathy target esterase | 170763470 |
| neuropeptide Y receptor type 5 | 157057095 |
| neuropilin 1 | 244792700 |
| neuropilin- and tolloid-like protein 1 | 251823964 |
| neurotrophic tyrosine kinase, receptor, type 2 isoform a | 68215970 |
| neurotrophic tyrosine kinase, receptor, type 3 isoform 1 | 33413412 |
| NT-3 growth factor receptor isoform b | 33413429 |
| nidogen 1 | 171543883 |
| nidogen 2 | 84370361 |
| Niemann Pick type C1 | 89242146 |
| NIPA-like domain containing 3 | 58037383 |
| nodal modulator 1 | 227908803 |
| occludin | 6679162 |
| olfactory receptor 1028 | 58801324 |
| olfactory receptor 109 | 22129189 |
| olfactory receptor 1094 | 22129573 |
| olfactory receptor 1118 | 148229648 |
| olfactory receptor 119 | 146261994 |
| olfactory receptor 1424 | 33239020 |
| olfactory receptor 174 | 121583641 |
| olfactory receptor 225 | 145699141 |
| olfactory receptor 310 | 268607639 |
| olfactory receptor 323 | 147901464 |
| olfactory receptor 411 | 289547748 |
| olfactory receptor 415 | 237874268 |
| olfactory receptor 470 | 33239110 |
| olfactory receptor 555 | 268607605 |
| olfactory receptor 561 | 22128785 |
| olfactory receptor 561 | 22128785 |
| olfactory receptor 600 | 268607577 |
| olfactory receptor 63 | 22128643 |
| olfactory receptor 675 | 58801454 |
| olfactory receptor 713 | 22128877 |
| olfactory receptor 805 | 22129529 |
| olfactory receptor 820 | 33239324 |
| olfactory receptor 873 | 49170044 |
| olfactory receptor 905 | 289629192 |
| olfactory receptor 984 | 33239012 |
| osteoblast differentiation promoting factor protein | 12963749 |
| otoferlin isoform 2 | 154240679 |
| pad-1-like isoform 1 | 62243808 |
| pancreatic lipase related protein 1 | 9256628 |
| patatin-like phospholipase domain containing 7 | 225007615 |
| PDZ domain containing 2 | 153791851 |
| PDZ domain containing 8 | 164698472 |
| pecanex homolog | 52632387 |
| pecanex-like 2 isoform 1 | 126352572 |
| pecanex-like 3 | 157743254 |
| pentraxin-related protein PTX3 precursor | 113930726 |
| peroxidasin | 268370173 |
| PEX5-related protein isoform 1 | 254588036 |
| phosphatidate cytidylyltransferase 2 | 20149726 |
| phosphatidylserine synthase 1 | 31560651 |
| phospholemman precursor | 37577137 |
| phospholipase A2, group IID | 7242177 |
| phospholipase A2, group VI | 8393978 |
| phosphoprotein associated with glycosphingolipid-enriched microdomains 1 | 31543456 |
| piggyBac transposable element derived 5 | 25777750 |
| placental prolactin-like protein N | 58037423 |
| plasmalemma vesicle associated protein | 255522953 |
| plasticity-related protein 2 | 283945516 |
| platelet/endothelial cell adhesion molecule 1 isoform 2 | 74024915 |
| pleckstrin homology domain-containing family B member 2 | 21704016 |
| pleckstrin homology domain-containing family H member 2 | 188497685 |
| plexin A2 | 113722113 |
| plexin A4 | 171543899 |
| plexin B1 | 225690610 |
| plexin D1 | 153792704 |
| poliovirus receptor | 28076965 |
| polycystic kidney disease 1-like isoform 1 | 78482609 |
| polycystic kidney disease 2-like 1 | 225543361 |
| polycystin 2 | 164519057 |
| polycystin-1 | 124487380 |
| polyductin | 126157466 |
| popeye domain containing 3 | 31745187 |
| pore forming protein-like | 148271095 |
| potassium channel tetramerisation domain containing 12b | 30425146 |
| potassium channel tetramerisation domain containing 13 | 27370096 |
| potassium channel, subfamily K, member 15 | 71892416 |
| potassium channel, subfamily K, member 3 | 33859576 |
| potassium channel, subfamily K, member 7 isoform a | 51944955 |
| potassium inwardly-rectifying channel J8 | 6680534 |
| potassium voltage-gated channel subfamily C member 3 | 133778992 |
| potassium voltage-gated channel KQT-like protein 2 isoform 1 | 54873639 |
| voltage-gated potassium channel subunit beta-1 | 148747467 |
| potassium voltage-gated channel subfamily A member 6 | 7305201 |
| potassium voltage-gated channel subfamily H member 8 | 73532782 |
| potassium voltage-gated channel, subfamily Q, member 1 | 70887795 |
| potassium voltage-gated channel, subfamily Q, member 3 | 282398106 |
| cadherin-9 | 169234947 |
| versican core protein isoform 1 | 124486955 |
| Down syndrome cell adhesion molecule-like protein 1 homolog | 124486901 |
| FAT tumor suppressor homolog 2 | 71725377 |
| GCN1 general control of amino-acid synthesis 1-like 1 | 112807186 |
| glutamate receptor, ionotropic, kainate 3 precursor | 124487364 |
| G protein-coupled receptor 179 | 124487045 |
| guanylate cyclase 2G precursor | 124487301 |
| HIV-1 induced protein HIN-1 | 124487191 |
| PREDICTED: hypothetical protein | 94390505 |
| interferon regulatory factor 2 binding protein 2 | 257196179 |
| PREDICTED: hypothetical protein | 51827457 |
| exocrine gland-secreting peptide 6 precursor | 294712521 |
| HHIP-like protein 1 precursor | 124249064 |
| protein CCSMST1 | 268607692 |
| hypothetical protein LOC230393 | 124487123 |
| HEAT repeat-containing protein 5B | 124487157 |
| PREDICTED: hypothetical protein LOC68725 | 269847632 |
| GLI pathogenesis-related 1 like 1 | 226443038 |
| adropin precursor | 197100326 |
| ATP-binding cassette, sub-family A (ABC1), member 12 | 225703056 |
| PREDICTED: hypothetical protein LOC75429 isoform 2 | 242247213 |
| von Willebrand factor A domain-containing protein 5B1 precursor | 198278486 |
| PREDICTED: hypothetical protein LOC78243 | 94396830 |
| immunoglobulin superfamily member 10 precursor | 242247270 |
| PREDICTED: kinase D-interacting substance of 220 kDa isoform 1 | 124487039 |
| motile sperm domain-containing protein 2 | 167234396 |
| PREDICTED: motile sperm domain containing 2 isoform 1 | 51772577 |
| neuregulin 1 | 124377986 |
| PREDICTED: neurobeachin-like 2 | 149260328 |
| NTPase KAP family P-loop domain-containing protein 1 | 254553317 |
| pappalysin 2 | 145699121 |
| PREDICTED: serine/threonine kinase 33 | 242118010 |
| collagen alpha-4(VI) chain precursor | 242247116 |
| apical endosomal glycoprotein | 238814398 |
| apolipoprotein B precursor | 161702988 |
| PREDICTED: similar to ATP-binding cassette, sub-family B, memb | 169234938 |
| basement membrane-specific heparan sulfate proteoglycan core protein | 183979966 |
| XVHEAT repeat family member 7B2 | 260593706 |
| PREDICTED: similar to calcyphosphine 2 | 94388920 |
| cartilage intermediate layer protein 2 | 168480100 |
| C-C chemokine receptor type 10 | 11024708 |
| PREDICTED: similar to Class II histocompatibility antigen, M b | 83013432 |
| collagen triple helix repeat-containing protein 1 precursor | 110625696 |
| PREDICTED: similar to Complement C4 precursor isoform 5 | 94409900 |
| PREDICTED: similar to complement component 7 precursor | 149266317 |
| contactin-associated protein like 5-3 precursor | 126116576 |
| von Willebrand factor C and EGF domain-containing protein precursor | 226442830 |
| contactin-associated protein like 5-3 precursor | 94403965 |
| FRAS1-related extracellular matrix protein 3 precursor | 268607688 |
| PREDICTED: similar to major histocompability complex Q1b | 149275071 |
| PREDICTED: similar to Histocompatibility 2, T region locus 3 | 149275145 |
| PREDICTED: similar to Ig gamma-2b chain membrane isoform 1 | 149263750 |
| PREDICTED: similar to interferon induced transmembrane protein | 94380332 |
| interleukin-31 precursor | 247301073 |
| PREDICTED: similar to KIAA1875 protein | 149266672 |
| PREDICTED: similar to KIAA1946 isoform 2 | 94366638 |
| laminin subunit alpha-3 | 226423935 |
| schlafen family member 14 | 260436908 |
| lipase member N precursor | 124249208 |
| PREDICTED: similar to Mas-related G-protein coupled receptor m | 82905791 |
| PREDICTED: similar to melanoma antigen family A, 10 | 83002172 |
| PREDICTED: similar to melanoma antigen, family B, 1 | 20984658 |
| PREDICTED: similar to MAGE-B3 | 94407646 |
| mucin-2 | 294610643 |
| myomesin-3 | 147906795 |
| PREDICTED: similar to N-acetylated alpha-linked acidic dipepti | 149250820 |
| PREDICTED: similar to nephrosis 1 homolog, nephrin | 149256957 |
| nesprin-2 | 145699091 |
| sialic acid binding Ig-like lectin 15 | 155372047 |
| PREDICTED: similar to NG5 protein | 94366216 |
| oxysterol-binding protein 1 | 133504509 |
| PREDICTED: similar to Oxysterol-binding protein 1 | 94406078 |
| fibrosin-1 | 241666423 |
| proprotein convertase subtilisin/kexin type 5 | 253314509 |
| paired basic amino acid cleaving system 4 | 242397450 |
| protocadherin-19 isoform a | 157426849 |
| PREDICTED: similar to putative membrane-bound dipeptidase 2 | 83002603 |
| vomeronasal receptor Vmn2r61 | 157311591 |
| PREDICTED: similar to putative pheromone receptor (Go-VN5) | 149268545 |
| vomeronasal receptor Vmn2r59 | 157311586 |
| vomeronasal receptor Vmn2r94 | 157151751 |
| calcium-transporting ATPase type 2C member 2 | 189339256 |
| NLR family, pyrin domain containing 12 | 257468329 |
| ring finger protein 180 | 110625861 |
| PREDICTED: similar to solute carrier organic anion transporter | 149255960 |
| PREDICTED: similar to sperm protein 3111 | 82998851 |
| testis-expressed sequence 10 protein | 124249335 |
| vomeronasal receptor Vmn2r4 | 157277946 |
| protein TANC2 | 124378026 |
| tyrosine-protein kinase RYK isoform 2 | 110681702 |
| PREDICTED: similar to vascular early response gene protein | 83030178 |
| motile sperm domain containing 4 | 254675302 |
| calcium channel, voltage-dependent, alpha 1I subunit | 157057194 |
| PREDICTED: similar to vomeronasal 2, receptor, 2 | 149274718 |
| vomeronasal 2, receptor 52 | 157384969 |
| vomeronasal 1 receptor 77 | 285399357 |
| WAP, kazal, immunoglobulin, kunitz and NTR domain-containing protein 1 precursor | 162462114 |
| PREDICTED: similar to Y51B11A.1 | 149262656 |
| zinc finger ZZ-type and EF-hand domain-containing protein 1 | 94390519 |
| sodium channel, voltage-gated, type III, alpha | 160707887 |
| sucrase-isomaltase | 124487275 |
| pregnancy specific glycoprotein 16 | 156938259 |
| pregnancy zone protein | 110347469 |
| pregnancy-specific glycoprotein 25 | 75832037 |
| pregnancy-specific glycoprotein 29 | 74959908 |
| prenylcysteine oxidase 1 | 13385294 |
| preproenkephalin 1 | 50950119 |
| procollagen, type I, alpha 1 | 34328108 |
| procollagen, type I, alpha 2 | 111120329 |
| procollagen, type IV, alpha 1 | 161484654 |
| procollagen, type IV, alpha 2 | 226437587 |
| procollagen, type IV, alpha 6 | 176866126 |
| procollagen, type IX, alpha 2 | 227116289 |
| procollagen, type V, alpha 3 | 8393173 |
| procollagen, type VII, alpha 1 | 115647999 |
| collagen alpha-1(XI) chain precursor | 124487346 |
| procollagen, type XII, alpha 1 | 111074529 |
| procollagen, type XVI, alpha 1 | 227500531 |
| procollagen, type XVII, alpha 1 | 226423931 |
| procollagen, type XVIII, alpha 1 | 40789282 |
| procollagen, type XXIV, alpha 1 | 116326001 |
| procollagen, type XXVII, alpha 1 | 116063546 |
| progestin and adipoQ receptor family member IV | 12963841 |
| prolactin-like protein E | 6679471 |
| proline arginine-rich end leucine-rich repeat | 229608920 |
| prolyl 4-hydroxylase, beta polypeptide | 42415475 |
| prostaglandin E receptor 4, subtype EP4 | 6679531 |
| prostaglandin F2 receptor negative regulator precursor | 50845420 |
| protease, serine, 15 | 116089322 |
| protease, serine, 25 | 254281222 |
| vitamin K-dependent protein C | 112421010 |
| serine/threonine-protein kinase D1 | 153945802 |
| protein O-mannosyltransferase 1 | 21553107 |
| protein tyrosine phosphatase, receptor type, B | 258613900 |
| protein tyrosine phosphatase, receptor type, C polypeptide-ass | 18640746 |
| protein tyrosine phosphatase, receptor type, T | 10946856 |
| liprin-beta-2 isoform 1 | 254588045 |
| proteolipid protein 1 | 23956058 |
| protocadherin 1 | 34328319 |
| protocadherin 12 | 120444908 |
| protocadherin 17 | 260763908 |
| protocadherin 18 | 170172572 |
| protocadherin 21 | 18700014 |
| protocadherin beta 11 | 148747868 |
| protocadherin beta 13 | 18087789 |
| protocadherin beta 15 | 18087793 |
| protocadherin gamma subfamily A, 4 | 148747499 |
| protocadherin gamma subfamily A, 6 | 18087763 |
| protocadherin gamma subfamily B, 4 | 18087737 |
| PTK7 protein tyrosine kinase 7 | 30425042 |
| putative membrane protein | 87116677 |
| pyruvate dehydrogenase E1 alpha 1 | 6679261 |
| RAB10, member RAS oncogene family | 7710086 |
| RAB15, member RAS oncogene family | 165377074 |
| RAB2, member RAS oncogene family | 10946940 |
| RAB21, member RAS oncogene family | 33859751 |
| RAB28, member RAS oncogene family | 58037191 |
| RAB33B, member of RAS oncogene family | 8394133 |
| RAB35, member RAS oncogene family | 37718983 |
| Rab38, member of RAS oncogene family | 21105857 |
| RAB7, member RAS oncogene family | 148747526 |
| rabphilin 3A | 16945962 |
| raft-linking protein | 33859813 |
| Ras and Rab interactor 1 | 21703974 |
| ras homolog gene family, member T2 | 22122457 |
| RAS-related C3 botulinum substrate 3 | 18875380 |
| receptor for egg jelly-like protein | 115583675 |
| recoverin | 6677693 |
| reelin | 117320554 |
| regulator of G protein signaling 7 | 190684703 |
| regulator of G-protein signaling 9 | 146134422 |
| relaxin 3 receptor 1 | 30520143 |
| reticulon 1 isoform RTN1-A | 31982561 |
| reticulon 4 isoform A | 34610235 |
| retinal degeneration, slow (retinitis pigmentosa 7) | 7110699 |
| rhomboid family 1 | 226437617 |
| rhomboid, veinlet-like 1 | 21450189 |
| rhomboid, veinlet-like 7 | 22122463 |
| ribophorin I | 282398108 |
| ribophorin II | 34996495 |
| RIKEN cDNA 0610013E23 | 21313294 |
| RIKEN cDNA 1110064P04 | 62530188 |
| palate lung and nasal carcinoma-like protein precursor | 258645128 |
| ring finger protein 128 | 283484004 |
| Ros1 proto-oncogene | 114326510 |
| roundabout homolog 4 | 27229187 |
| Rous sarcoma oncogene isoform 2 | 70794809 |
| RuvB-like protein 1 | 9790083 |
| ryanodine receptor 1, skeletal muscle | 145046267 |
| ryanodine receptor 2, cardiac | 124430578 |
| Sad1 and UNC84 domain containing 1 | 29243924 |
| zeta-sarcoglycan | 171543881 |
| sarcoma antigen NY-SAR-41 | 58037313 |
| SPARC-related modular calcium-binding protein 1 isoform 2 | 226246620 |
| secreted phosphoprotein 1 | 165932360 |
| sema domain, immunoglobulin domain (Ig), short basic domain, s | 31543683 |
| sema domain, transmembrane domain (TM), and cytoplasmic domain | 40385869 |
| sema domain, transmembrane domain (TM), and cytoplasmic domain | 6755464 |
| semaphorin 3A | 31543681 |
| semaphorin 3C | 46048361 |
| semaphorin 3D | 282847344 |
| semaphorin 3E | 113199777 |
| semaphorin 4D | 49274623 |
| seminal vesicle antigen-like 3 | 56710338 |
| protein SERAC1 isoform 2 | 161169008 |
| shadow of prion protein | 34147163 |
| shugoshin-like 1 | 13386294 |
| sialyltransferase 8 F | 22003888 |
| sidekick homolog 1 | 164518930 |
| skeletal muscle receptor tyrosine kinase isoform 4 precursor | 82533047 |
| SLAM family member 7 | 31541977 |
| SLIT and NTRK-like family, member 5 isoform 2 | 56699418 |
| small conductance calcium-activated potassium channel protein | 158854044 |
| small inducible cytokine subfamily E, member 1 | 126012517 |
| sodium channel, nonvoltage-gated, type I, alpha | 257796261 |
| sodium channel, voltage-gated, type IV, alpha polypeptide | 134948032 |
| excitatory amino acid transporter 4 | 6678003 |
| solute carrier family 12 member 9 | 281371456 |
| solute carrier family 13 member 1 | 171543829 |
| monocarboxylate transporter 14 | 41281606 |
| solute carrier family 16, member 3 | 13507630 |
| solute carrier family 2, facilitated glucose transporter member 3 | 261862282 |
| solute carrier family 2, facilitated glucose transporter member 1 | 165377226 |
| solute carrier family 2, facilitated glucose transporter member 5 | 31543728 |
| solute carrier family 2, member 9 isoform 2 | 22094111 |
| solute carrier family 2, member 9 isoform a | 156231031 |
| solute carrier family 22 member 14 | 83627689 |
| solute carrier family 22 member 7 | 254939683 |
| calcium-binding mitochondrial carrier protein Aralar2 isoform 1 | 7657583 |
| calcium-binding mitochondrial carrier protein Aralar1 | 27369581 |
| phosphate carrier protein, mitochondrial precursor | 19526818 |
| solute carrier family 25, member 28 | 21553115 |
| sulfate anion transporter 1 | 170650677 |
| solute carrier family 26 (sulfate transporter), member 2 | 6681233 |
| sodium/nucleoside cotransporter 2 | 27370488 |
| equilibrative nucleoside transporter 1 | 12584968 |
| 4F2 cell-surface antigen heavy chain isoform b | 238637279 |
| solute carrier family 30 (zinc transporter), member 6 | 31559966 |
| zinc transporter 9 | 125660458 |
| solute carrier family 34 (sodium phosphate), member 2 | 66793411 |
| proton-coupled amino acid transporter 3 | 262231848 |
| solute carrier family 38, member 4 | 31543737 |
| solute carrier family 39 (zinc transporter), member 14 | 47059049 |
| electrogenic sodium bicarbonate cotransporter 1 isoform a | 133922580 |
| anion exchange protein 4 | 27370244 |
| sodium bicarbonate cotransporter 3 | 117320529 |
| sodium/glucose cotransporter 1 | 261824023 |
| sodium-coupled monocarboxylate transporter 2 isoform 1 | 51491856 |
| sodium- and chloride-dependent neutral and basic amino acid transporter B(0+) | 254281193 |
| large neutral amino acids transporter small subunit 1 | 31982764 |
| solute carrier family 8 (sodium/calcium exchanger), member 2 | 22507355 |
| sodium/hydrogen exchanger 4 | 45238583 |
| solute carrier organic anion transporter family, member 1a5 | 18700000 |
| solute carrier organic anion transporter family, member 6c1 | 58037373 |
| VPS10 domain-containing receptor SorCS1 | 158966696 |
| sortilin-related VPS10 domain containing receptor 3 | 23956144 |
| sorting and assembly machinery component 50 homolog | 30519943 |
| paraplegin | 148539988 |
| sperm-specific protein Izumo | 65301151 |
| ST3 beta-galactoside alpha-2,3-sialyltransferase 4 | 31543703 |
| stabilin 1 | 154240684 |
| sterol O-acyltransferase 1 | 84619697 |
| sulfatase 2 | 61656167 |
| suppression of tumorigenicity 14 (colon carcinoma) | 7363445 |
| surfactant associated protein D | 6677921 |
| synaptotagmin IX | 31543799 |
| synaptotagmin VII gamma isoform | 160948571 |
| synaptotagmin-like 4 | 7305099 |
| exophilin-5 | 257796249 |
| synoviolin 1 | 258547102 |
| syntaxin 2 | 170172516 |
| syntaxin 7 | 31560462 |
| taste receptor, type 2, member 124 | 46309599 |
| taste receptor, type 2, member 130 | 40255297 |
| T-cell lymphoma invasion and metastasis 1 | 225543222 |
| tectorin alpha | 239787867 |
| testicular serine protease 1 | 6678293 |
| thioredoxin domain containing 11 isoform 1 | 28077061 |
| thrombopoietin | 6678341 |
| thrombospondin 2 | 239787900 |
| thrombospondin 3 | 239915968 |
| thromboxane A synthase 1, platelet | 79750133 |
| cytokine receptor-like factor 2 isoform 2 | 257900526 |
| lamina-associated polypeptide 2 isoform alpha | 121949760 |
| thyroglobulin | 124430576 |
| thyroid peroxidase | 6678417 |
| tight junction protein ZO-1 isoform 1 | 254675277 |
| tight junction protein 4 (peripheral) | 21311861 |
| Tmc5 protein | 32441288 |
| toll-like receptor 9 | 157057166 |
| tolloid-like protein 1 precursor | 117414180 |
| trace amine-associated receptor 9 | 58082063 |
| transferrin receptor | 11596855 |
| transforming growth factor, beta 3 | 225637540 |
| nesprin-3 isoform beta | 111607451 |
| transient receptor potential cation channel, subfamily C, member 1 | 6755885 |
| transient receptor potential cation channel, subfamily M, member 2 | 261878536 |
| transient receptor potential cation channel, subfamily M, member 3d | 78214532 |
| transient receptor potential cation channel, subfamily M, member 7 | 256773285 |
| transient receptor potential cation channel, subfamily M, member 8 | 19527416 |
| transient receptor potential cation channel, subfamily V, member 1 | 74315350 |
| ransient receptor potential cation channel subfamily V member 3 | 266458389 |
| transient receptor potential cation channel, subfamily V, member 6 | 28376639 |
| translocase of outer mitochondrial membrane 40 homolog-like | 83921605 |
| translocase of outer mitochondrial membrane 70 homolog A | 27552760 |
| transmembrane and coiled coil domains 3 | 78771620 |
| transmembrane and coiled-coil domains 2 | 30725710 |
| transmembrane protease, serine 2 | 34328226 |
| transmembrane protease, serine 8 (intestinal) | 24496768 |
| transmembrane protein 10 | 23943844 |
| transmembrane protein 16B | 209862776 |
| transmembrane protein 16F | 40254290 |
| transmembrane protein 2 | 76253922 |
| transmembrane protein 20 | 30425262 |
| transmembrane protein 33 isoform 1 | 22267448 |
| transmembrane protein 39a | 27754069 |
| transmembrane protein 43 | 21311891 |
| transmembrane protein 44 | 100817930 |
| transmembrane protein 48 | 28077005 |
| transmembrane protein 55A | 21312268 |
| transmembrane protein 57 | 40254350 |
| Meckelin | 240255631 |
| antigen peptide transporter 2 | 239915963 |
| triggering receptor expressed on myeloid cells 2 | 13994125 |
| tryptase 4 | 11055972 |
| tsukushi | 67972425 |
| tumor necrosis factor (ligand) superfamily, member 10 | 6678431 |
| tumor necrosis factor receptor superfamily, member 21 | 30519885 |
| tumor rejection antigen gp96 | 6755863 |
| tweety 2 | 68989251 |
| type IIc Na+/Pi-cotransporter | 224994177 |
| tyrosine kinase receptor 1 | 144446133 |
| ubiquitin-activating enzyme E1, Chr X | 6678483 |
| UDP glucuronosyltransferase 2 family, polypeptide B35 | 27370342 |
| UDP-Gal:betaGlcNAc beta 1,3-galactosyltransferase, polypeptide | 157266277 |
| UDP-GalNAc:polypeptide N-acetylgalactosaminyltransferase-like | 32401429 |
| UDP-GlcNAc:betaGal beta-1,3-N-acetylglucosaminyltransferase | 31542175 |
| UDP-glucose ceramide glucosyltransferase | 7106443 |
| UDP-N-acetyl-alpha-D-galactosamine:polypeptide N-acetylgalacto | 7657112 |
| polypeptide N-acetylgalactosaminyltransferase 3 | 162951828 |
| unc-5 homolog B | 85861262 |
| Unc-51 like kinase 1 | 40254402 |
| unc-84 homolog A | 29789243 |
| unc-84 homolog B | 168693641 |
| uncoupling protein 3 (mitochondrial, proton carrier) | 6678495 |
| upregulated during skeletal muscle growth 5 | 77404294 |
| usherin | 145699097 |
| vesicle-associated membrane protein, associated protein A | 94721328 |
| vesicle-associated membrane protein, associated protein B and | 31543940 |
| vesicular membrane protein p24 | 6678577 |
| voltage gated channel like 1 | 123173782 |
| voltage-dependent calcium channel gamma-5 subunit | 21687006 |
| voltage-dependent calcium channel gamma-8 subunit | 21687186 |
| voltage-gated sodium channel type V alpha | 84875498 |
| vomeronasal 1 receptor, E1 | 21717703 |
| vomeronasal 1 receptor, H2 | 33238868 |
| vomeronasal 1 receptor, H4 | 21717747 |
| vomeronasal 1 receptor, I6 | 282165735 |
| Vpr-binding protein | 82617569 |
| VPS10 domain receptor protein SORCS 2 | 124301217 |
| WD repeat and HMG-box DNA binding protein 1 | 40254224 |
| WD repeat domain 18 | 83649741 |
| wingless-related MMTV integration site 7A | 31543960 |
| Wolfram syndrome 1 protein homolog | 6755997 |
| zinc finger protein 318 isoform 1 | 157823950 |
| zinc finger, DHHC domain containing 5 | 21450253 |
